# Cardiac Troponin Thresholds in Children and Young Adults: A Multi-Center Cohort Study

**DOI:** 10.1093/jalm/jfaf205

**Published:** 2026-01-21

**Authors:** Alexander J F Thurston, Eirik Å Røys, Ragnhild Røysland, Øyvind Skadberg, Fabienne Decrue, Dorien M Kimenai, Nicholas L Mills, Kristin M Aakre

**Affiliations:** BHF Centre for Cardiovascular Science, The University of Edinburgh, Edinburgh, United Kingdom; Department of Clinical Science, University of Bergen, Bergen, Norway; Department of Medical Biochemistry and Pharmacology, Haukeland University Hospital, Bergen, Norway; Multidisciplinary Laboratory Medicine and Medical Biochemistry, Division of Diagnostics and Technology, Akershus University Hospital, Lørenskog, Norway; Institute of Clinical Medicine, Faculty of Medicine, University of Oslo, Oslo, Norway; Laboratory of Medical Biochemistry, Stavanger University Hospital, Stavanger, Norway; BHF Centre for Cardiovascular Science, The University of Edinburgh, Edinburgh, United Kingdom; BHF Centre for Cardiovascular Science, The University of Edinburgh, Edinburgh, United Kingdom; BHF Centre for Cardiovascular Science, The University of Edinburgh, Edinburgh, United Kingdom; Usher Institute, University of Edinburgh, Edinburgh, United Kingdom; Department of Clinical Science, University of Bergen, Bergen, Norway; Department of Medical Biochemistry and Pharmacology, Haukeland University Hospital, Bergen, Norway; Department of Heart Disease, Haukeland University Hospital, Bergen, Norway

## Abstract

**Background:**

The role of high-sensitivity cardiac troponin (cTn) assays for children and young adults is uncertain, and no guidance is available on diagnostic thresholds. This study evaluates the effect of applying pediatric compared to adult upper reference limits (URLs) for cTn.

**Methods:**

We carried out a retrospective multicenter international cohort study of consecutive children and young adults (1 day to 18 years) undergoing cTn I or T testing at 4 tertiary care hospitals in Norway and Scotland, United Kingdom, from 2013 to 2023. Myocardial injury was classified using the adult sex-specific 99th percentile URL, a pediatric sex-specific 99th percentile, and a pediatric sex-specific 97.5th percentile. Diagnoses of myocarditis were obtained from the Norwegian Patient Register and the Scottish Morbidity Record.

**Results:**

In total, 9833 (46.6% female) children and young adults underwent cTn testing. Applying the adult sex-specific 99th percentile, 1771 (18.0% [95% CI, 17.3%–18.8%]) had myocardial injury compared with 1762 (17.9% [95% CI, 17.2%–18.7%]) using a pediatric 99th percentile. In contrast, applying a pediatric sex-specific 97.5th percentile would identify 2261 (23.0% [95% CI, 22.2%–23.8%]) with myocardial injury (a 28% relative increase). Infants had a higher frequency of myocardial injury than those 1–18 years old (1035/1104; 93.8% [95% CI, 92.2%–95.1%] vs 1226/8729; 14.0% [95% CI, 13.3%–14.8%] using pediatric sex-specific 97.5th percentile, *P* < 0.001). Testing for cTn increased over the study period (τ = 0.42, *P* < 0.001).

**Conclusions:**

The use of pediatric-specific 97.5th percentile URLs for cTn would increase classification of myocardial injury in children and young adults. The clinical implications of this are uncertain and require further study given cTn testing has increased over the last decade.

IMPACT STATEMENT Recent expert statements highlight limited data on cardiac troponin (cTn) testing in children and young adults. We conducted a multicenter international cohort study of 9833 patients to assess the impact of adopting pediatric-specific upper reference limits (URLs) for high-sensitivity cTn assays. Using the pediatric 97.5th percentile as the URL increased the proportion classified with myocardial injury compared with manufacturers’ adult thresholds. These findings have important implications for healthcare resource use and may inform future clinical guidance on interpreting troponin results in pediatric populations.

## INTRODUCTION

Cardiac troponin (cTn) is released into the circulation following cardiomyocyte stress. When the cTn concentration increases above the upper reference limit of healthy individuals this is defined as myocardial injury (1). In adults, the underlying pathophysiological mechanisms and the diagnostic and prognostic role of cTn have been investigated for decades with multiple guidelines available to guide care (2–5). In contrast, there is less published data available on cTn testing in children and young adults and no recommendations on indications for testing or appropriate thresholds to guide practice (6, 7). While myocardial infarction is very rare in children, the recognition of myocardial injury is important in conditions presenting in childhood, including myocarditis, cardiomyopathies, and congenital heart diseases (8–11). Furthermore, the use of cTn testing in children is likely to have increased during the severe acute respiratory syndrome coronavirus-2 (SARS-CoV-2) pandemic and subsequent vaccination campaign due to widespread public and clinical concerns about cardiac involvement and myocarditis (12, 13).

Differentiating normal from abnormal values is the basis of detection of underlying cardiac pathology with cTn testing. Whilst the conduct of reference range studies in adults to define appropriate sex-specific 99th percentile upper reference limits (URLs) is now recommended by international guidelines (14) and is mandatory for regulatory approval of cTn assays, manufacturers are not required to determine separate reference limits in children and young adults. Large general population studies in adults demonstrate that the URL varies for all cTn assays and is influenced by age and sex (15–17). Previous studies have evaluated 99th percentile values of pediatric cohorts, typically with a single high-sensitivity cardiac troponin (hs-cTn) assay, with variable sample sizes, approach to stratification by age and sex, and statistical handling of outlying values; published values are therefore heterogenous and have not been widely adopted in practice (18–23).

McEvoy et al. recently reported findings from 5695 healthy children and young adults between 1 to 18 years of age participating in the National Health and Nutrition Examination Survey (NHANES) (24). Pediatric sex- and age-specific 99th percentiles were derived for 4 hs-cTn assays. The authors also suggested that the 97.5th percentile may be an appropriate limit to define normal cTn values in children and young adults. This approach would be consistent with the majority of other biomarker assays and less affected by outliers, whereas in adults the 99th percentile is selected primarily to maximize specificity for a diagnosis of myocardial infarction, which is rarely the reason for cTn testing in children and young adults (6, 25).

This study aims to determine whether the use of pediatric-specific reference intervals for cTn would influence the proportion of children and young adults identified with myocardial injury compared to manufacturer-recommended thresholds derived from adult populations. Furthermore, it evaluates trends in pediatric cTn testing frequency and identification of myocardial injury over the past decade.

## MATERIALS AND METHODS

### Study Design

A retrospective multicenter international cohort study was conducted at 4 tertiary-care hospitals in Norway and Scotland, United Kingdom: Akershus University Hospital, Haukeland University Hospital, Stavanger University Hospital, and the Royal Hospital for Children and Young People in Edinburgh. The study was approved by the Regional Committee for Medical and Health Research Ethics in Bergen, Norway (ID number 685657) and by the Regional Ethics Committee and Caldicott Guardian in Edinburgh, United Kingdom (reference number: 22/NS/0093). As demographic data and cTn concentrations were obtained from routine laboratory information systems, deidentified, and stored within a trusted research environment (DataLoch, Edinburgh, United Kingdom) or dedicated servers for research approved and secured by the Health Care Trusts in Norway, individual participant level consent was not required. Fully anonymized data on diagnoses of myocarditis and pericarditis were extracted from the Norwegian Patient Registry (26) and the Scottish Morbidity Record (27).

### Study Population

The study population consisted of consecutive children and young adults ≤18 years of age who underwent at least one hs-cTn measurement between January 1, 2013, and December 31, 2023, as recorded in the laboratory information systems of the four participating hospitals. Unique individuals were enrolled based on their first recorded cTn measurement. Children and young adults were stratified by age as infants (<1 year), children (1 to 12 years), and adolescents (>12 years) and by sex, and no exclusion criteria were applied.

### High-Sensitivity Cardiac Troponin Assays

cTn T was measured using a high-sensitivity assay on a Cobas e602 or e801 from Roche Diagnostics. The assay has a limit of blank (LOB) of 3 ng/L, limit of detection (LOD) of 5 ng/L, limit of quantification (LOQ) at 6 ng/L, and adult uniform and sex-specific 99th percentile URLs of 14 ng/L, 9 ng/L (females), and 16 ng/L (males), respectively. The measurement range is 3 to 10 000 ng/L (28, 29) The analytical within-series coefficient of variation (CV_A_) is <10% at 14 ng/L.

cTn I was measured using a high-sensitivity assay on an ARCHITECT i2000 and Alinity system from Abbott Diagnostics. This assay has a LoB of 1.0 ng/L, a LoD of 1.6 ng/L, a LoQ of 1.3 ng/L, and uniform and sex-specific 99th percentile URLs of 26 ng/L, 16 ng/L (females), and 34 ng/L (males), respectively (29, 30). The measurement range is 1 to 50 000 ng/L. The CV_A_ is <10% at 4.7 ng/L.

For cTn measurements that were below the local limit for reporting, we assigned a value half the specified limit. Testing at the Royal Hospital for Children and Young People in Edinburgh switched from a hs-cTnI assay to a hs-cTnT assay on October 27, 2021. No other site switched assay during the study period.

### Classification of Myocardial Injury

To evaluate the impact of applying pediatric or adult URLs on the classification of myocardial injury, we used the presentation cTn concentration and applied thresholds from 3 sources: the local thresholds used in clinical practice at each participating site, the adult sex-specific 99th percentile URLs recommended by the assay manufacturer, and the pediatric sex-specific 99th and 97.5th percentile URLs derived from the NHANES cohort (24). We calculated the number of children and young adults who would have been reclassified with or without myocardial injury by switching from the adult to pediatric thresholds. The youngest participant in the NHANES derivation cohort was 12 months old, and therefore we conducted a sensitivity analysis of the classification of myocardial injury excluding those under one year old.

### Trends in Testing and Diagnosis

To evaluate trends in cTn testing, the number of cTn tests was compared to creatinine by site annually and quarterly. We examined case rates of myocarditis and pericarditis relative to cTn testing across the same period. The number of hospital discharge diagnoses of myocarditis or pericarditis was determined using the International Classification of Disease 10th edition (ICD-10) codes I30, I31, I32, I40, I41, and I51.4 (31). Contemporary European guidance on the diagnosis of myocarditis advocates criteria combining a clinical presentation suggestive of myocarditis with one or more of the typical electrocardiographic changes, detection of myocardial injury through elevated cardiac troponin concentrations, functional or structural abnormalities on cardiac imaging, or typical appearances on cardiac magnetic resonance, with coronary angiography and endomyocardial biopsy for confirmation in selected cases (32). Due to data-protection policies to mitigate risk of re-identification, annual or quarterly case counts <5 were censored by the registry (Norway) or converted to density plots (United Kingdom) before disclosure from the trusted research environment.

### Statistical Analysis

Sites contributed data aggregated by sex (female or male) and by age group (<1 years, 1 to 12 years and >12 years) per quarter from their respective secure data environments for a pooled analysis. Cardiac troponin concentrations were reported as median (25th–75th percentile) by site. Categorical variables were reported as number and proportion (*n* [%]) and were analyzed by site and across the cohort in the pooled analysis. Stratification was performed by assay (hs-cTnI or hs-cTnT), sex, age group, and site.

For binomial proportions with myocardial injury, 95% CIs were calculated by the exact method and groups were compared with χ^2^ tests or Fisher exact tests. Trends in the volume of cTn testing and proportion of tests above the local URL were assessed using the Mann–Kendall test from 2014 to 2023 (when data was available for all sites). We performed a sensitivity analysis excluding the years 2020 to 2022 to account for the effects of the SARS-CoV-2 pandemic. Trends in testing at individual sites were evaluated across the complete study period for each site. Hypothesis testing was two-sided at a significance level of 0.05.

All analyses were performed in R version 4.4.1 (R Foundation for Statistical Computing). This study followed Strengthening the Reporting of Observational Studies in Epidemiology (STROBE) reporting guidelines.

## RESULTS

### Characteristics

A total of 9833 children and young adults underwent cTn testing across the four study sites over the study period (Table 1). The cohort was balanced by sex, with 53.4% male (*n* = 5255) and 46.6% female (*n* = 4578). The majority of those undergoing cTn testing were adolescents ≥12 years old (66.9%), followed by children 1 to 12 years old (21.8%) and infants <1 year old (11.2%). The sex balance and proportion of adolescents and children undergoing testing was similar across sites, with the greatest variation observed in the use of cTn testing in infants, ranging from 4.4% to 15.1%.

**Table 1.** Baseline characteristics of children and young adults undergoing cardiac troponin testing across sites.

	All sites		Site A		Site B		Site C		Site D	
Country			Norway		Norway		United Kingdom		Norway	
Study period	2013–2023		2013–2023		2013–2023		2014–2023		2013–2023	
Number	9833		3702	—	3333	—	1558	—	1240	—
Sex	—	—	—	—	—	—	—	—	—	—
Female	4578	(46.6%)	1732	(46.8%)	1551	(46.5%)	724	(46.5%)	571	(46.0%)
Male	5255	(53.4%)	1970	(53.2%)	1782	(53.5%)	834	(53.5%)	669	(54.0%)
Age	—	—	—	—	—	—	—	—	—	—
Less than 1 year	1104	(11.2%)	366	(9.9%)	503	(15.1%)	69	(4.4%)	166	(13.4%)
1 to 12 years	2146	(21.8%)	1217	(32.9%)	529	(15.9%)	204	(13.1%)	196	(15.8%)
Over 12 years	6583	(66.9%)	2119	(57.2%)	2301	(69.0%)	1285	(82.5%)	878	(70.8%)
Creatinine test in study period	236 261	—	57 307	—	36 069	—	109 707	—	33 178	—

### Classification of Myocardial Injury

**Fig. 1.**
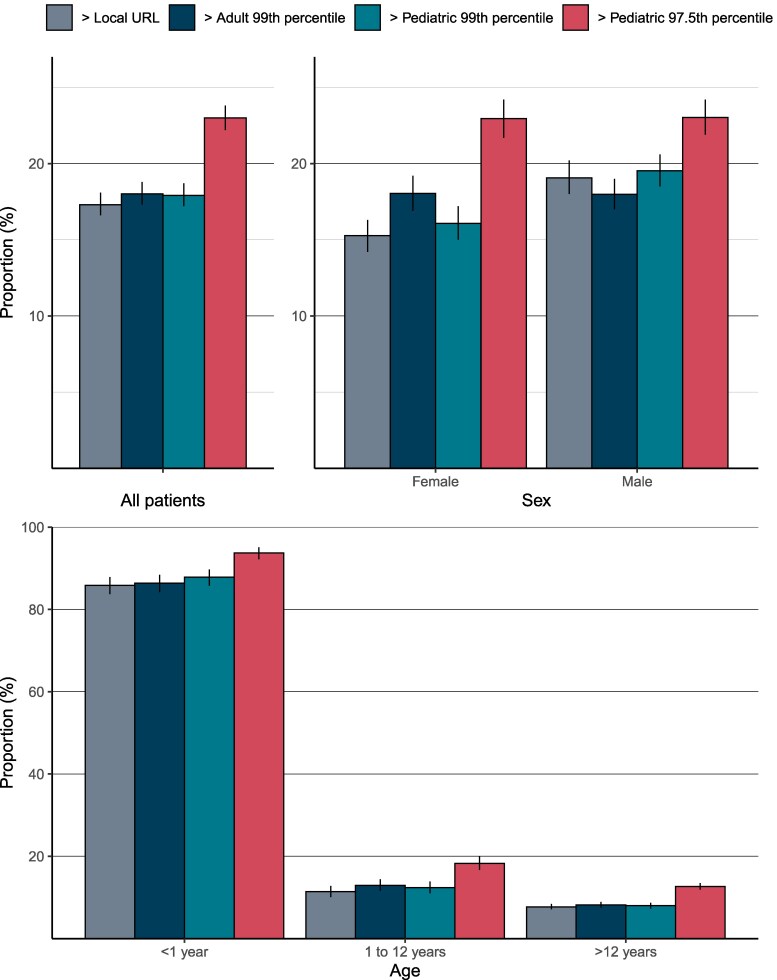
Proportion (%) of cardiac troponin tests above local laboratory reference limits (gray), adult sex-specific 99th percentile (blue), pediatric sex-specific 99th percentile (teal), and pediatric sex-specific 97.5th percentile (red). Error bars represent 95% confidence intervals. Proportions are shown for the whole cohort (upper left panel) and grouped by sex (upper right panel) and age range (lower panel).

Among all 9833 children and young adults undergoing cTn testing, 1701 (17.3% [95% CI, 16.6%–18.1%]) had values that exceeded the local laboratory URL (Table 2) and were identified as having myocardial injury in practice. Applying the manufacturer-recommended adult sex-specific 99th percentiles, 1771 (18.0% [95% CI, 17.3%–18.8%]) of the children and young adults undergoing cTn testing would be classified as having myocardial injury. In comparison, applying pediatric sex-specific 99th percentiles would reclassify 81 (0.8%) children and young adults with myocardial injury, and 90 (0.9%) without myocardial injury, resulting in 1762 (17.9% [95% CI, 17.2%–18.7%]) with myocardial injury. In contrast, applying the pediatric sex-specific 97.5th percentiles identified 2261 (23.0% [95% CI, 22.2%–23.8%]) with myocardial injury: a 5% absolute and 28% relative increase compared to the adult sex-specific 99th percentile (Fig. 1, Table 3, Supplemental Table 1).

**Table 2.** Assay characteristics and reference limits across the four study sites.

	Site A	Site B	Site C	Site C	Site D
	—	—	(until October 26, 2021)	(after October 27, 2021)	—
Country	Norway	Norway	UK	UK	Norway
Assay	hs-cTnT	hs-cTnT	hs-cTnI	hs-cTnT	hs-cTnI
Instrument	e801	e801	ARCHITECT	e801	ARCHITECT/Alinity^a^
Assay manufacturer	Roche	Roche	Abbott	Roche	Abbott
Limit of detection	3 ng/L	3 ng/L	1.9 ng/L	3 ng/L	1.9 ng/L
CV <10%	13 ng/L	13 ng/L	3 ng/L	13 ng/L	3 ng/L
Lower reporting limit^b^	3 ng/L	3 ng/L	1 ng/L	3 ng/L	2 ng/L
Results below reporting limit	1430	1135	672	151	89
Local URL	—	—	—	—	—
Female	14 ng/L	14 ng/L	>16 years: 16 ng/L	>16 years: 9 ng/L	15 ng/L
			≤16 years: 26 ng/L	≤16 years: 14 ng/L	
Male	14 ng/L	14 ng/L	>16 years: 34 ng/L	>16 years: 16 ng/L	30 ng/L
			≤16 years: 26 ng/L	≤16 years: 14 ng/L	
Adult URL^c^ (manufacturer)	—	—	—	—	—
Female—99th percentile	9 ng/L	9 ng/L	16 ng/L	9 ng/L	16 ng/L
Male—99th percentile	16 ng/L	16 ng/L	34 ng/L	16 ng/L	34 ng/L
NHANES pediatric cohort	—	—	—	—	—
Female—99th percentile	12 ng/L	12 ng/L	17 ng/L	12 ng/L	17 ng/L
Male—99th percentile	15 ng/L	15 ng/L	16 ng/L	15 ng/L	16 ng/L
Female—97.5th percentile	6 ng/L	6 ng/L	5 ng/L	6 ng/L	5 ng/L
Male—97.5th percentile	11 ng/L	11 ng/L	9 ng/L	11 ng/L	9 ng/L

^a^Stavanger switched from an ARCHITECT instrument to an Alinity instrument on June 28, 2019.

^b^These data are the most recent lower reporting limits for each laboratory. Historical values are presented in Supplemental Table 3.

^c^Upper reference limits (URLs) are derived from three sources: (*a*) local URLs, (*b*) manufacturer-reported adult sex-specific 99th percentiles, and (*c*) sex-specific 99th and 97.5th percentiles from the NHANES healthy pediatric cohort (24).

**Table 3.** Number of children and young adults with hs-cTn values above each decision threshold, grouped by assay, sex, and age group. Percentages are given in parentheses with 95% confidence intervals in brackets.

	All sites	hs-cTnI	hs-cTnT
Total	9833	2264	7569
Above local reference limit	1701 (17.3% [16.6–18.1%])	285 (12.6% [11.2–14.0%])	1416 (18.7% [17.8–19.6%])
Above sex-specific adult 99th percentile	1771 (18.0% [17.3–18.8%])	281 (12.4% [11.1–13.8%])	1490 (19.7% [18.8–20.6%])
Above sex-specific pediatric 99th percentile	1762 (17.9% [17.2–18.7%])	339 (15.0% [13.5–16.5%])	1423 (18.8% [17.9–19.7%])
Above sex-specific pediatric 97.5th percentile	2261 (23.0% [22.2–23.8%])	434 (19.2% [17.6–20.9%])	1827 (24.1% [23.2–25.1%])
Sex	—	—	—
Female	4578	1043	3535
Above local reference limit	699 (15.3% [14.2–16.3%])	108 (10.4% [8.6–12.4%])	591 (16.7% [15.5–18.0%])
Above sex-specific adult 99th percentile	826 (18.0% [16.9–19.2%])	114 (10.9% [9.1–13.0%])	712 (20.1% [18.8–21.5%])
Above sex-specific pediatric 99th percentile	736 (16.1% [15.0–17.2%])	111 (10.6% [8.8–12.7%])	625 (17.7% [16.4–19.0%])
Above sex-specific pediatric 97.5th percentile	1051 (23.0% [21.7–24.2%])	163 (15.6% [13.5–18.0%])	888 (25.1% [23.7–26.6%])
Male	5255	1221	4034
Above local reference limit	1002 (19.1% [18.0–20.2%])	177 (14.5% [12.6–16.6%])	825 (20.5% [19.2–21.7%])
Above sex-specific adult 99th percentile	945 (18.0% [17.0–19.0%])	167 (13.7% [11.8–15.7%])	778 (19.3% [18.1–20.5%])
Above sex-specific pediatric 99th percentile	1026 (19.5% [18.5–20.6%])	228 (18.7% [16.5–21.0%])	798 (19.8% [18.6–21.0%])
Above sex-specific pediatric 97.5th percentile	1210 (23.0% [21.9–24.2%])	271 (22.2% [19.9–24.6%])	939 (23.3% [22.0–24.6%])
Age	—	—	—
Less than 1 year	1104	202	902
Above local reference limit	948 (85.9% [83.7–87.9%])	132 (65.3% [58.3–71.9%])	816 (90.5% [88.4–92.3%])
Above sex-specific adult 99th percentile	954 (86.4% [84.2–88.4%])	126 (62.4% [55.3–69.1%])	828 (91.8% [89.8–93.5%])
Above sex-specific pediatric 99th percentile	970 (87.9% [85.8–89.7%])	152 (75.2% [68.7–81.0%])	818 (90.7% [88.6–92.5%])
Above sex-specific pediatric 97.5th percentile	1035 (93.8% [92.2–95.1%])	173 (85.6% [80.0–90.2%])	862 (95.6% [94.0–96.8%])
1 to 12 years	2146	281	1865
Above local reference limit	245 (11.4% [10.1–12.8%])	33 (11.7% [8.2–16.1%])	212 (11.4% [10.0–12.9%])
Above sex-specific adult 99th percentile	278 (13.0% [11.6–14.4%])	36 (12.8% [9.1–17.3%])	242 (13.0% [11.5–14.6%])
Above sex-specific pediatric 99th percentile	266 (12.4% [11.0–13.9%])	42 (14.9% [11.0–19.7%])	224 (12.0% [10.6–13.6%])
Above sex-specific pediatric 97.5th percentile	392 (18.3% [16.7–20.0%])	53 (18.9% [14.5–23.9%])	339 (18.2% [16.5–20.0%])
Over 12 years	6583	1781	4802
Above local reference limit	508 (7.7% [7.1–8.4%])	120 (6.7% [5.6–8.0%])	388 (8.1% [7.3–8.9%])
Above sex-specific adult 99th percentile	539 (8.2% [7.5–8.9%])	119 (6.7% [5.6–7.9%])	420 (8.7% [8.0–9.6%])
Above sex-specific pediatric 99th percentile	526 (8.0% [7.3–8.7%])	145 (8.1% [6.9–9.5%])	381 (7.9% [7.2–8.7%])
Above sex-specific pediatric 97.5th percentile	834 (12.7% [11.9–13.5%])	208 (11.7% [10.2–13.3%])	626 (13.0% [12.1–14.0%])

### Stratification by Assay

More children and young adults were identified with myocardial injury in clinical practice when tested with a hs-cTnT assay compared to a hs-cTnI assay (1416/7569; 18.7% [95% CI, 17.8%–19.6%] vs 285/2264; 12.6% [95% CI, 11.2%–14.0%], *P* < 0.001). This difference was consistent across all thresholds evaluated (Table 3). Switching from adult to pediatric sex-specific 99th percentile thresholds would reclassify more males with injury using a hs-cTnI assay compared to the hs-cTnT assay (61 [5.0%] vs 20 [0.5%], *P* < 0.001) but would reclassify fewer females without myocardial injury (3 [0.1%] vs 87 [1.1%], *P* < 0.001) (Supplemental Table 2).

### Stratification by Sex and Age

More male than female children and young adults were identified with myocardial injury in clinical practice using the local URLs (1002/5255; 19.1% [95% CI, 18.0%–20.2%] vs 699/4578; 15.3% [95% CI, 14.2%–16.3%], *P* < 0.001). In contrast, the application of sex-specific pediatric 97.5th percentiles identified an identical proportion of male and female children with myocardial injury (1210/5255; 23.0% [95% CI, 21.9%–24.2%] vs 1051/4578; 23.0% [95% CI, 21.7%–24.2%], *P* = 0.96). The proportions with myocardial injury were not balanced using the sex-specific pediatric 99th percentile, which identified more males than females with myocardial injury (1026/5255; 19.5% [95% CI, 18.5%–20.6%] vs 736/4578; 16.1% [95% CI, 15.0%–17.2%], *P* < 0.001).

The majority of infants had myocardial injury identified in clinical practice using the local URLs (948/1104; 85.9% [95% CI, 83.7%–87.9%]), whereas 245/2146 children (11.4% [95% CI, 10.1%–12.8%]) and 508/6583 adolescents (7.7% [95% CI, 7.1%–8.4%]) had myocardial injury (*P* < 0.001). This observation was consistent by sex, across all sites and for both hs-cTnI and hs-cTnT assays (Fig. 1, Table 3, Supplemental Table 3).

### Sensitivity Analysis in Children and Adolescents

In a sensitivity analysis excluding infants (*n* = 1104), 753 of 8729 (8.6% [95% CI, 8.0%–9.2%]) children and adolescents had cTn concentrations above local URLs. Applying adult 99th percentiles and pediatric 99th percentiles would classify 817 (9.4% [95% CI, 8.8%–10.0%]) and 792 (9.1% [95% CI, 8.5%–9.7%]) with myocardial injury, respectively. By comparison, the pediatric 97.5th percentile would identify 1226 (14.0% [95% CI, 13.3%–14.8%]) children and adolescents with myocardial injury, an absolute increase of 4.9% and relative increase of 55% compared to the pediatric 99th percentile (Supplemental Table 5).

### Frequency and Trends in Cardiac Troponin Testing

Across the study period, sites conducted around 230 cTn tests per year (9833 across 43 site-years). Testing for cTn was performed in 1 in 24 children and young adults who underwent creatinine testing (9833/236 261) (Table 1).

**Fig. 2.**
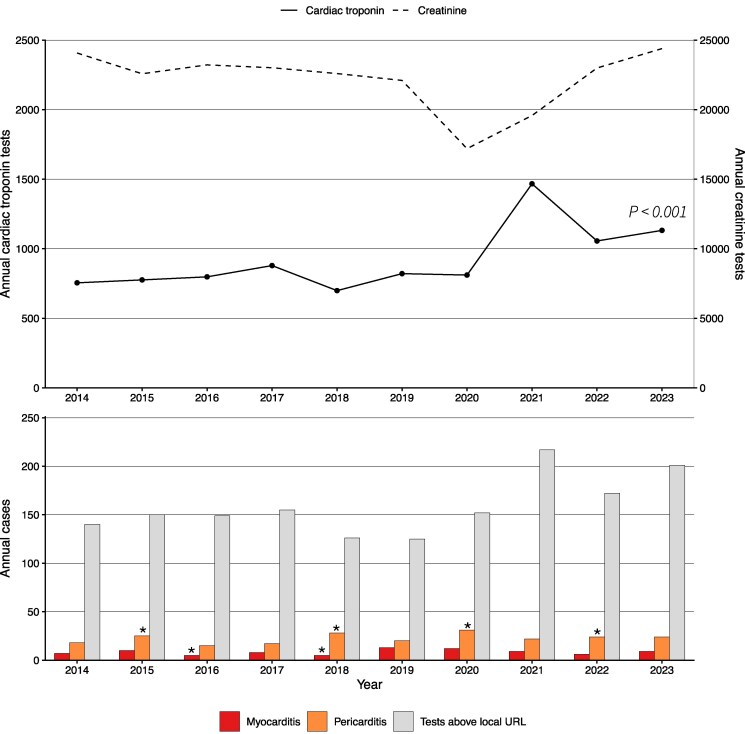
Annual number of cardiac troponin tests and creatinine tests at all sites. *P* values indicate the significance of trends in test numbers. The lower panel depicts annual numbers of tests exceeding the local upper reference limit (URL), cases of myocarditis (red), and pericarditis (orange) across all sites. Where annual counts are marked by an asterisk, at least one site reported <5 annual cases rather than an absolute value to protect confidentiality of individuals. True counts may therefore be lower.

Testing for cTn increased over the study period (τ = 0.42, *P* < 0.001), with a notable rise in 2021 that coincided with the COVID-19 pandemic and a marked increase in diagnoses of COVID-19 within the regions of each site (Fig. 2, Supplemental Figs. 2–4). In a sensitivity analysis excluding the years of the COVID-19 pandemic (2020 to 2022), this positive trend remained (τ = 0.33, *P* = 0.02). While the number of cTn tests performed increased, no trend was observed in the proportion of children and young adults identified with myocardial injury in practice using the local URL (τ = −0.16, *P* = 0.14). Similar findings were observed for all thresholds evaluated (Supplemental Fig. 5).

### Myocarditis and Pericarditis

The diagnosis of myocarditis or pericarditis in children and young adults was infrequent across all sites during the study period (Fig. 2; Supplemental Figs. 2 and 3). Despite the increase in cTn testing, no corresponding rise in the diagnosis of myocarditis or pericarditis was observed. No relationship was observed between myopericarditis diagnoses and cTn testing, cases of COVID-19 or SARS-CoV-2 vaccinations where data were available (Supplemental Figs. 2 and 3).

## DISCUSSION

This study quantifies the potential impact of adopting pediatric sex-specific cTn thresholds on the identification of myocardial injury in practice in nearly 10 000 consecutive children and young adults. Switching from adult 99th percentiles to pediatric 99th percentiles would reclassify less than 1% of those undergoing testing either with or without myocardial injury. In contrast, applying pediatric 97.5th percentiles would increase the proportion of children and young adults with myocardial injury from 18% to 23%. In clinical practice, cTn testing in this population is infrequent (33–35) and this increase represents a small absolute number of additional children and young adults identified with myocardial injury (on average 11 per year at each site). However, the clinical impact of increasing the identification of myocardial injury in children and young adults remains uncertain. Cost analyses indicate that elevated cTn results in children precipitate a large number of investigations (8) and could cause anxiety and reduce quality of life for the child, parents, and extended family, and may even increase risk if resulting in invasive cardiac testing or general anesthesia.

The NHANES cohort is the largest study in which cTn concentrations have been determined in healthy children and young adults. Pediatric sex-specific 99th and 97.5th percentile values have previously been derived for the hs-cTnT assay in the Canadian Laboratory Initiative on Pediatric Reference Intervals (CALIPER) cohort, with close agreement with the NHANES study (97.5th percentiles: female 6 ng/L and male 11 ng/L in both studies; 99th percentiles: female 11 ng/L [CALIPER] vs 12 ng/L [NHANES], male 14 ng/L [CALIPER] vs 15 ng/L [NHANES]) (18). Furthermore, the NHANES study evaluated 4 different hs-cTn assays within the same cohort, enhancing confidence that reference values will perform consistently across different assays.

In their derivation of these thresholds, McEvoy et al. observed that confidence intervals around 97.5th percentile values were narrower than for 99th percentiles, and point estimates were less variable according to the statistical approach to outliers, in keeping with previous observations (24, 25). In our study of real-world testing, the proportion of male and female patients identified with myocardial injury was more consistent using pediatric 97.5th percentile thresholds than pediatric 99th percentiles, which may be a manifestation of this increased statistical precision. The 99th percentile is recommended in adult patients to mitigate concerns around false positives in the detection of myocardial infarction with high-sensitivity assays, but this is rarely the indication for testing in the pediatric setting (14). Moreover, recruiting large cohorts of healthy children for reference studies is inherently challenging; hence, use of a threshold that can be estimated with more precision and stability might seem appealing, although an increased statistical certainty needs to be balanced against potential clinical drawbacks including unnecessary investigations and patient anxiety.

Pediatric cTn testing increased over the 10-year study period, although the proportion with myocardial injury remained similar. Most sites increased testing during the COVID-19 pandemic with a corresponding fall in proportion of results exceeding the local URL. The overall incidence of pericarditis and myocarditis was low and stable at all sites regardless of rates of COVID-19 or vaccination (where data were available).

The majority of infants had cTn values above all thresholds we examined, consistent with reference interval studies that have shown infants have several-fold higher concentrations than older children (18, 20–22, 28, 36–39). No specific cutoffs for children below 1 year were used by site laboratories or available from manufacturers, perhaps understandably as URLs below 1 year are often based on few individuals and large between-study differences exist in the reported thresholds. The NHANES cohort did not include any infants under 1 year, and our data show that the thresholds from this study could not be extrapolated meaningfully to infants in clinical practice. If these thresholds were adopted in practice, laboratories and assay manufacturers should include guidance to caution users that they do not apply to infants, for whom higher cTn concentrations are not necessarily an indicator of cardiac disease.

As in adults, the predominant indication for cTn testing in children is chest pain (8, 40), although the most frequently diagnosed condition is myocarditis (41, 42). Other common pediatric indications for testing include trauma, poisoning, and drug overdose, but cTn measurement also has a role in the assessment of those with congenital heart disease, arrythmia, heart failure, or cardiac surgery (8, 35, 40, 43, 44). Similarly to the adult population, cTn may be used for monitoring of cancer treatment or in the evaluation of myocardial involvement in systemic or rheumatological conditions (44). One study using contemporary cTn measurements reported that a cardiac diagnosis was made in 60% of children with an increased cTn concentration compared to 12% with normal concentrations (33), whereas in an emergency department cohort tested with high-sensitivity assays, 75% with an elevated hs-cTnT had a cardiac cause (35). Contrastingly, in a small single-center study evaluating pediatric 97.5th percentiles, only 21% received a cardiac diagnosis (45). Correspondingly in our study, the volume of myocarditis cases was much lower than the number of tests exceeding any of the threshold values. There is a tension between the value of hs-cTn testing for the detection of cardiac conditions in this population (35, 42) and the cost implications and burden on children and families from follow-up investigations triggered by the identification of myocardial injury (8), particularly if the pre-test probability of cardiac disease is low. Careful selection of patients for testing is important regardless of the reference limit adopted, and the need remains for additional research to shape guidance from pediatric cardiology and laboratory societies on the clinical indications for testing and interpretation of results.

### Strength and Limitations

This study included all children and young adults undergoing cTn testing across four sites in two countries, using both hs-cTnT and hs-cTnI assays, allowing for robust evaluation of the impact of alternative decision thresholds in contemporary clinical practice. We compared testing frequency and myocardial injury to the most common clinical endpoint expected in children undergoing cTn testing, myocarditis (41, 44), and the concurrent incidence of COVID-19 in the pediatric population.

There are important limitations to our findings which must be considered. Data on the clinical indication for testing was not available and would add important context to our findings. Moreover, we did not have data on participant ethnicity and thus caution must be applied when generalizing our findings to other healthcare settings. Due to the mandatory requirement for data sourced from national patient registers to be fully anonymized, individual test results could not be linked to specific cardiac diagnoses, and absolute case numbers were often too low to be disclosed without risk of identifying individuals. Therefore, neither the diagnostic accuracy of cTn testing in children nor the consequences at an individual level could be investigated. Furthermore, for the same reason, patient age at testing was only disclosed as an integer in years at some sites, which prevented a granular analysis of individuals across the first days and months of life. The methodology for assessing regional incidence of COVID-19 differed between UK (individual hospitalizations) and Norwegian sites (regional cases confirmed by laboratory PCR or antigen testing, aggregated by patients 1 day to 19 years old); thus, we could not formally evaluate the relationship between COVID-19 and cTn testing in a pooled analysis.

## CONCLUSIONS

In children and young adults, the use of pediatric sex-specific 97.5th percentiles for the interpretation of cTn would significantly increase the recognition of myocardial injury, whereas the use of sex-specific pediatric and adult 99th percentiles identify a similar proportion with myocardial injury. None of these thresholds are applicable to infants less than 1 year old. The clinical implications of this are uncertain and require further study given cTn testing has increased in this population over the last 10 years.

## Supplementary Material

jfaf205_Supplementary_Data

## Data Availability

Norwegian sites: The study utilized de-identified individual-level data obtained from Norwegian hospital laboratory systems and the Norwegian Patient Registry (NPR) held in a trusted research environment. Access to these data is regulated under Norwegian law and requires approval from the Regional Committees for Medical and Health Research Ethics (REK) and the local data protection officer. Due to data protection regulations, the data sets are not publicly available but can be accessed upon reasonable request, provided that the necessary ethical approvals and data protection agreements may be obtained. UK site: The study used linked, de-identified, and aggregated individual patient level data from routine electronic healthcare data sources that is held in a trusted research environment by DataLoch (https://dataloch.org/). The study data and analysis code can be accessed by individuals who have undertaken the necessary governance training on application to DataLoch.

## Notes

**Nonstandard Abbreviations:** cTn, cardiac troponin; URL, upper reference limit; SARS-CoV-2, severe acute respiratory syndrome coronavirus-2; hs-cTn, high-sensitivity cardiac troponin; NHANES, National Health and Nutrition Examination Survey; LOB, limit of blank; LOD, limit of detection; LOQ, limit of quantification; CV_A_, analytical within-series coefficient of variation; ICD, International Classification of Disease; STROBE, Strengthening the Reporting of Observational Studies in Epidemiology; CALIPER, Canadian Laboratory Initiative on Pediatric Reference Intervals.

**Author Contributions**: *The corresponding author takes full responsibility that all authors on this publication have met the following required criteria of eligibility for authorship: (a) significant contributions to the conception and design, acquisition of data, or analysis and interpretation of data; (b) drafting or revising the article for intellectual content; (c) final approval of the published article; and (d) agreement to be accountable for all aspects of the article thus ensuring that questions related to the accuracy or integrity of any part of the article are appropriately investigated and resolved. Nobody who qualifies for authorship has been omitted from the list.*

Alexander Thurston (Data curation-Equal, Formal analysis-Lead, Project administration-Equal, Visualization-Equal, Writing—original draft-Equal, Writing—review & editing-Equal), Eirik Røys (Data curation-Equal, Formal analysis-Equal, Project administration-Equal, Writing—original draft-Equal), Ragnhild Røysland (Data curation-Equal, Project administration-Equal, Writing—review & editing-Equal), Oyvind Skadberg (Data curation-Equal, Project administration-Equal, Writing—review & editing-Equal), Fabienne Decrue (Methodology-Equal, Writing—review & editing-Equal), Dorien Kimenai (Conceptualization-Equal, Methodology-Equal, Supervision-Equal, Writing—review & editing-Equal), Nicholas Mills (Conceptualization-Equal, Funding acquisition-Equal, Methodology-Equal, Writing—review & editing-Equal), and Kristin Aakre (Conceptualization-Equal, Funding acquisition-Equal, Methodology-Equal, Supervision-Equal, Writing—original draft-Equal, Writing—review & editing-Equal)

**Authors’ Disclosures or Potential Conflicts of Interest**: *Upon manuscript submission, all authors completed the author disclosure form.*

**Research Funding:** A.J.F. Thurston is supported by a Clinical Research Training Fellowship from the British Heart Foundation (FS/CRTF/25/24735). E.Å. Røys is supported by a PhD grant from the Helse Vest (F-12821). D.M. Kimenai is supported by an Intermediate Basic Science Research Fellowship and Research Excellence Award from the British Heart Foundation (FS/IBSRF/23/25161, RE/24/130012). N.L. Mills is supported by a Chair Award, Programme Grant, and Research Excellence Award from the British Heart Foundation (CH/F/21/90010, RG/20/10/34966, RE/24/130012). K.M. Aakre is supported by a grant from the Helse Vest (F-12976).

**Disclosures:** A.J.F. Thurston has received a research grant from Roche Diagnostics outside the submitted work, paid to institution. D.M. Kimenai has received honoraria from Roche Diagnostics and Abbott Diagnostics. N.L. Mills has received honoraria or consultancy from Roche Diagnostics, Abbott Laboratories, and Siemens Healthineers over the last 36 months. K.M. Aakre has served on advisory boards for Roche Diagnostics, Radiometer, Siemens Healthineers, and SpinChip, received consultant honoraria form CardiNor; lecturing honoraria from Siemens Healthineers, Roche Diagnostics, Mindray, and Snibe Diagnostics; and research grants from Siemens Healthineers and Roche Diagnostics; is an Associate Editor of *Clinical Biochemistry* and Chair of the IFCC Committee of Clinical Application of Cardiac Bio-markers.

**Role of Sponsor:** The funding organizations played no role in the design of study, choice of enrolled patients, review and interpretation of data, preparation of manuscript, or final approval of manuscript.
