# Supplementary material for: Cardiac Troponin Thresholds in Children and Young Adults: A Multi-Center Cohort Study
Source: J Appl Lab Med. 2026 Jan 21;11(3):554–68. doi: 10.1093/jalm/jfaf205 (PMC13141673; doi:10.1093/jalm/jfaf205)

**Supplementary material**

**Cardiac troponin thresholds in children and young adults: a multi-centre cohort study**

Alexander J F Thurston^1*^, Eirik Å. Røys^2,3*^, Ragnhild Røysland^4,5^, Øyvind Skadberg^6^, Fabienne Decrue^1^, Dorien M Kimenai^1^, Nicholas L Mills^1,7*^, Kristin M Aakre^2,3,8*^

^1^ BHF Centre for Cardiovascular Science, The University of Edinburgh, Edinburgh, UK
^2^ Department of Clinical Science, University of Bergen, Bergen, Norway
^3^ Department of Medical Biochemistry and Pharmacology, Haukeland University Hospital, Bergen, Norway
^4^ Multidisciplinary Laboratory Medicine and Medical Biochemistry, Division of Diagnostics and Technology, Akershus University Hospital, Lørenskog, Norway ^5^ Institute of Clinical Medicine, Faculty of Medicine, University of Oslo, Oslo, Norway
^6^ Laboratory of Medical Biochemistry, Stavanger University Hospital, Stavanger, Norway
^7^ Usher Institute, University of Edinburgh, Edinburgh, UK
^8^ Department of Heart Disease, Haukeland University Hospital, Bergen, Norway

^*^both authors contributed equally to this paper

**Contents**

**STROBE Statement – page 2
Supplementary methods – page 5
Supplementary table 1 – page 6
Supplementary table 2 – page 7
Supplementary table 3 – page 8
Supplementary table 4- page 11
Supplementary table 5 – page 12
Supplementary figure 1 – page 13
Supplementary figure 2 – page 14
Supplementary figure 3 – page 15
Supplementary figure 4 – page 16
Supplementary figure 5 – page 17**

**STROBE Statement**—Checklist of items that should be included in reports of ***cohort studies***

|  | Item No | Recommendation | Page |
| --- | --- | --- | --- |
| **Title and abstract** | 1 | (*a*) Indicate the study’s design with a commonly used term in the title or the abstract | 1 |
|  |  | (*b*) Provide in the abstract an informative and balanced summary of what was done and what was found | 3 |
| Introduction | | |  |
| Background/rationale | 2 | Explain the scientific background and rationale for the investigation being reported | 4 |
| Objectives | 3 | State specific objectives, including any prespecified hypotheses | 5 |
| Methods | | |  |
| Study design | 4 | Present key elements of study design early in the paper | 5 |
| Setting | 5 | Describe the setting, locations, and relevant dates, including periods of recruitment, exposure, follow-up, and data collection | 5-6 |
| Participants | 6 | (*a*) Give the eligibility criteria, and the sources and methods of selection of participants. Describe methods of follow-up | 6 |
|  |  | (*b*) For matched studies, give matching criteria and number of exposed and unexposed | NA |
| Variables | 7 | Clearly define all outcomes, exposures, predictors, potential confounders, and effect modifiers. Give diagnostic criteria, if applicable | 7 |
| Data sources/ measurement | 8* | For each variable of interest, give sources of data and details of methods of assessment (measurement). Describe comparability of assessment methods if there is more than one group | 6-7 |
| Bias | 9 | Describe any efforts to address potential sources of bias | 8 |
| Study size | 10 | Explain how the study size was arrived at | 6 |
| Quantitative variables | 11 | Explain how quantitative variables were handled in the analyses. If applicable, describe which groupings were chosen and why | 7-8 |
| Statistical methods | 12 | (*a*) Describe all statistical methods, including those used to control for confounding | 7-8 |
|  |  | (*b*) Describe any methods used to examine subgroups and interactions | 8 |
|  |  | (*c*) Explain how missing data were addressed | NA |
|  |  | (*d*) If applicable, explain how loss to follow-up was addressed | NA |
|  |  | (*e*) Describe any sensitivity analyses | 7-8 |
| Results | | |  |
| Participants | 13* | (a) Report numbers of individuals at each stage of study—eg numbers potentially eligible, examined for eligibility, confirmed eligible, included in the study, completing follow-up, and analysed | Lines 198-204, Table 1 |
|  |  | (b) Give reasons for non-participation at each stage | NA |
|  |  | (c) Consider use of a flow diagram | NA |
| Descriptive data | 14* | (a) Give characteristics of study participants (eg demographic, clinical, social) and information on exposures and potential confounders | 8 |
|  |  | (b) Indicate number of participants with missing data for each variable of interest | NA |
|  |  | (c) Summarise follow-up time (eg, average and total amount) | NA |
| Outcome data | 15* | Report numbers of outcome events or summary measures over time | 9 |
| Main results | 16 | (*a*) Give unadjusted estimates and, if applicable, confounder-adjusted estimates and their precision (eg, 95% confidence interval). Make clear which confounders were adjusted for and why they were included | 9 |
|  |  | (*b*) Report category boundaries when continuous variables were categorized | 7 |
|  |  | (*c*) If relevant, consider translating estimates of relative risk into absolute risk for a meaningful time period | NA |
| Other analyses | 17 | Report other analyses done—eg analyses of subgroups and interactions, and sensitivity analyses | 9-10 |
| Discussion | | |  |
| Key results | 18 | Summarise key results with reference to study objectives | 11-12 |
| Limitations | 19 | Discuss limitations of the study, taking into account sources of potential bias or imprecision. Discuss both direction and magnitude of any potential bias | 13-14 |
| Interpretation | 20 | Give a cautious overall interpretation of results considering objectives, limitations, multiplicity of analyses, results from similar studies, and other relevant evidence | 14 |
| Generalisability | 21 | Discuss the generalisability (external validity) of the study results | 13 |
| Other information | | |  |
| Funding | 22 | Give the source of funding and the role of the funders for the present study and, if applicable, for the original study on which the present article is based | 16 |

*Give information separately for exposed and unexposed groups.

**Note:** An Explanation and Elaboration article discusses each checklist item and gives methodological background and published examples of transparent reporting. The STROBE checklist is best used in conjunction with this article (freely available on the Web sites of PLoS Medicine at http://www.plosmedicine.org/, Annals of Internal Medicine at http://www.annals.org/, and Epidemiology at http://www.epidem.com/). Information on the STROBE Initiative is available at http://www.strobe-statement.org.

**Supplementary methods**

Cases, hospitalizations and vaccinations for COVID-19

To assess the potential impact of the COVID-19 pandemic on cTn testing, the number of reported pediatric diagnoses within the regions of each tertiary-care site were determined from 2020-2022. In Norway, these data were obtained from the Notification System for Infectious Diseases of the Norwegian Institute of Public Health^1^, which includes laboratory-confirmed diagnoses in the community and hospital aggregated from 0-19 years of age. In Scotland, these were determined from the Scottish Morbidity Record reflecting only those admitted to hospital with a diagnosis of COVID-19 between 0-18 years of age. Additional data on SARS-CoV-2 vaccination for children and young adults (0-18 years) were available in Scotland from Public Health Scotland.

References

1. Folkehelseinstituttet. Notification System for Infectious Diseases <https://statistikk.fhi.no>. Accessed 20/3/2025, 2025.

**Supplementary Table 1:** Number and proportion of children & young adults reclassified with or without myocardial injury by sex-specific pediatric 99^th^ and 97·5^th^ percentile thresholds, in comparison to manufacturer’s adult sex-specific 99^th^ percentile thresholds.

|  | >adult URL | Reclassified | |
| --- | --- | --- | --- |
|  |  | with injury | *without injury* |
| Total | 1771/9833 (18·0%) | - | - |
| > pediatric sex-specific 99^th^ percentile | - | 81 (0·8%) | *90 (0·9%)* |
| > pediatric sex-specific 97·5^th^ percentile | - | 490 (5·0%) | - |
| Female | 826/4578 (18·0%) | - | - |
| > pediatric sex-specific 99^th^ percentile | - | - | *90 (2·0%)* |
| > pediatric sex-specific 97·5^th^ percentile | - | 225 (4·9%) | - |
| Male | 945/5255 (18·0%) | - | - |
| > pediatric sex-specific 99^th^ percentile | - | 81 (1·5%) | - |
| > pediatric sex-specific 97·5^th^ percentile | - | 265 (5·0%) | - |

**Supplementary Table 2:** Number and proportion of children & young adults reclassified with or without myocardial injury by sex-specific pediatric 99^th^ and 97·5^th^ percentile thresholds, in comparison to manufacturer’s adult sex-specific 99^th^ percentile thresholds, according to cardiac troponin assay.

|  | **hs-cTnI** | | | **hs-cTnT** | | |
| --- | --- | --- | --- | --- | --- | --- |
|  | >adult URL | Reclassified | | >adult URL | Reclassified | |
|  |  | with injury | *without* *injury* |  | with injury | *without* *injury* |
| Total | 281/2264 (12·4%) | - | - | 1490/7569 (19·7%) | - | - |
| > pediatric 99th percentile | - | 61 (2·7%) | *3 (0·1%)* | - | 20 (0·3%) | *87 (1·1%)* |
| > pediatric 97·5th percentile | - | 153 (6·8%) | - | - | 337 (4·4%) | - |
| Female | 114/1043 (10·4%) | - | - | 712/3535 (20·1%) | - | - |
| > pediatric 99th percentile | - | - | *3 (0·3%)* | - | - | *87 (2·5%)* |
| > pediatric 97·5th percentile | - | 49 (4·7%) | - | - | 176 (5%) | - |
| Male | 167/1221 (13·7%) | - | - | 778/4034 (19·3%) | - | - |
| > pediatric 99th percentile | - | 61 (5·0%) | - | - | 20 (0·5%) | - |
| > pediatric 97·5th percentile | - | 104 (8·5%) | - | - | 161 (4%) | - |

**Supplementary Table 3:** Differences in cTn concentration [25^th^-75^th^ percentile] and number (proportion [95% confidence intervals]) above each threshold grouped by site, age group and sex.

|  | **All Sites** | **Site A** | **Site B** |
| --- | --- | --- | --- |
| Country |  | Norway | Norway |
| Study period | 2013-2023 | 2013-2023 | 2013-2023 |
| Total | 9833 | 3702 | 3333 |
| Above local reference limit | 1701 (17·3% [16·6–18·1%]) | 566 (15·3% [14·1–16·5%]) | 781 (23·4% [22·0–24·9%]) |
| Above sex-specific adult 99^th^ percentile | 1771 (18·0% [17·3–18·8%]) | 605 (16·3% [15·2–17·6%]) | 813 (24·4% [22·9–25·9%]) |
| Above sex-specific pediatric 99^th^ percentile | 1762 (17·9% [17·2–18·7%]) | 575 (15·5% [14·4–16·7%]) | 782 (23·5% [22·0–24·9%]) |
| Above sex-specific pediatric 97·5^th^ percentile | 2261 (23·0% [22·2–23·8%]) | 776 (21·0% [19·7–22·3%]) | 952 (28·6% [27·0–30·1%]) |
| Median troponin concentration [IQR] |  | 5 ng/L [2-7 ng/L] | 5 ng/L [2-12 ng/L] |
| Sex |  |  |  |
| Female | 4578 | 1732 | 1551 |
| Above local reference limit | 699 (15·3% [14·2–16·3%]) | 241 (13·9% [12·3–15·6%]) | 326 (21·0% [19·0–23·1%]) |
| Above sex-specific adult 99^th^ percentile | 826 (18·0% [16·9–19·2%]) | 299 (17·3% [15·5–19·1%]) | 381 (24·6% [22·4–26·8%]) |
| Above sex-specific pediatric 99^th^ percentile | 736 (16·1% [15·0–17·2%]) | 261 (15·1% [13·4–16·8%]) | 339 (21·9% [19·8–24·0%]) |
| Above sex-specific pediatric 97·5^th^ percentile | 1051 (23·0% [21·7–24·2%]) | 390 (22·5% [20·6–24·6%]) | 450 (29·0% [26·8–31·3%]) |
| Median troponin concentration [IQR] |  | 4 ng/L [2-6 ng/L] | 4 ng/L [2-9 ng/L] |
| Male | 5255 | 1970 | 1782 |
| Above local reference limit | 1002 (19·1% [18·0–20·2%]) | 325 (16·5% [14·9–18·2%]) | 455 (25·5% [23·5–27·6%]) |
| Above sex-specific adult 99^th^ percentile | 945 (18·0% [17·0–19·0%]) | 306 (15·5% [14·0–17·2%]) | 432 (24·2% [22·3–26·3%]) |
| Above sex-specific pediatric 99^th^ percentile | 1026 (19·5% [18·5–20·6%]) | 314 (15·9% [14·3–17·6%]) | 443 (24·9% [22·9–26·9%]) |
| Above sex-specific pediatric 97·5^th^ percentile | 1210 (23·0% [21·9–24·2%]) | 386 (19·6% [17·9–21·4%]) | 502 (28·2% [26·1–30·3%]) |
| Median troponin concentration [IQR] |  | 5 ng/L [3-8 ng/L] | 5 ng/L [4-15 ng/L] |
| Age |  |  |  |
| Less than 1 year | 1104 | 366 | 503 |
| Above local reference limit | 948 (85·9% [83·7–87·9%]) | 313 (85·5% [81·5–89·0%]) | 479 (95·2% [93·0–96·9%]) |
| Above sex-specific adult 99^th^ percentile | 954 (86·4% [84·2–88·4%]) | 318 (86·9% [83·0–90·2%]) | 486 (96·6% [94·6–98·0%]) |
| Above sex-specific pediatric 99^th^ percentile | 970 (87·9% [85·8–89·7%]) | 314 (85·8% [81·8–89·2%]) | 480 (95·4% [93·2–97·1%]) |
| Above sex-specific pediatric 97·5^th^ percentile | 1035 (93·8% [92·2–95·1%]) | 342 (93·4% [90·4–95·8%]) | 493 (98·0% [96·4–99·0%]) |
| Median troponin concentration [IQR] |  | 49 ng/L [26-112 ng/L] | 124 ng/L [64-261 ng/L] |
| 1 to 12 years | 2146 | 1217 | 529 |
| Above local reference limit | 245 (11·4% [10·1–12·8%]) | 123 (10·1% [8·5–11·9%]) | 67 (12·7% [10·0–15·8%]) |
| Above sex-specific adult 99^th^ percentile | 278 (13·0% [11·6–14·4%]) | 144 (11·8% [10·1–13·8%]) | 75 (14·2% [11·3–17·4%]) |
| Above sex-specific pediatric 99^th^ percentile | 266 (12·4% [11·0–13·9%]) | 133 (10·9% [9·2–12·8%]) | 70 (13·2% [10·5–16·4%]) |
| Above sex-specific pediatric 97·5^th^ percentile | 392 (18·3% [16·7–20·0%]) | 208 (17·1% [15·0–19·3%]) | 97 (18·3% [15·1–21·9%]) |
| Median troponin concentration [IQR] |  | 4 ng/L [2-6 ng/L] | 4 ng/L [2-6 ng/L] |
| Over 12 years | 6583 | 2119 | 2301 |
| Above local reference limit | 508 (7·7% [7·1–8·4%]) | 130 (6·1% [5·2–7·2%]) | 235 (10·2% [9·0–11·5%]) |
| Above sex-specific adult 99^th^ percentile | 539 (8·2% [7·5–8·9%]) | 143 (6·7% [5·7–7·9%]) | 252 (11·0% [9·7–12·3%]) |
| Above sex-specific pediatric 99^th^ percentile | 526 (8·0% [7·3–8·7%]) | 128 (6·0% [5·1–7·1%]) | 232 (10·1% [8·9–11·4%]) |
| Above sex-specific pediatric 97·5^th^ percentile | 834 (12·7% [11·9–13·5%]) | 226 (10·7% [9·4–12·1%]) | 362 (15·7% [14·3–17·3%]) |
| Median troponin concentration [IQR] |  | 4 ng/L [2-5 ng/L] | 4 ng/L [2-6 ng/L] |

|  | **Site C (hs-cTnI)** | | **Site C (hs-cTnT)** | | **Site D** |
| --- | --- | --- | --- | --- | --- |
| Country | UK | | UK | | Norway |
| Study period | 2014-2021 | | 2021-2023 | | 2013-2023 |
| Total | 1024 | | 534 | | 1240 |
| Above local reference limit | 85 (8·3% [6·7–10·2%]) | | 69 (12·9% [10·2–16·1%]) | | 200 (16·1% [14·1–18·3%]) |
| Above sex-specific adult 99^th^ percentile | 84 (8·2% [6·6–10·1%]) | | 72 (13·5% [10·7–16·7%]) | | 197 (15·9% [13·9–18·0%]) |
| Above sex-specific pediatric 99^th^ percentile | 106 (10·4% [8·6–12·4%]) | | 66 (12·4% [9·7–15·5%]) | | 233 (18·8% [16·7–21·1%]) |
| Above sex-specific pediatric 97·5^th^ percentile | 134 (13·1% [11·1–15·3%]) | | 99 (18·5% [15·3–22·1%]) | | 300 (24·2% [21·8–26·7%]) |
| Median troponin concentration [IQR] | 1 ng/L [1-2 ng/L] | | 4 ng/L [2-7 ng/L] | | 2 ng/L [2-7 ng/L] |
| Sex |  | |  | |  |
| Female | 472 | | 252 | | 571 |
| Above local reference limit | 27 (5·7% [3·8–8·2%]) | | 24 (9·5% [6·2–13·8%]) | | 81 (14·2% [11·4–17·3%]) |
| Above sex-specific adult 99^th^ percentile | 33 (7·0% [4·9–9·7%]) | | 32 (12·7% [8·9–17·5%]) | | 81 (14·2% [11·4–17·3%]) |
| Above sex-specific pediatric 99^th^ percentile | 32 (6·8% [4·7–9·4%]) | | 25 (9·9% [6·5–14·3%]) | | 79 (13·8% [11·1–16·9%]) |
| Above sex-specific pediatric 97·5^th^ percentile | 47 (10·0% [7·4–13·0%]) | | 48 (19·0% [14·4–24·4%]) | | 116 (20·3% [17·1–23·9%]) |
| Median troponin concentration [IQR] | 1ng/L [1-1ng/L] | | 3ng/L [2-5ng/L] | | 2ng/L [2-3ng/L] |
| Male | 552 | | 282 | | 669 |
| Above local reference limit | 58 (10·5% [8·1–13·4%]) | | 45 (16·0% [11·9–20·8%]) | | 119 (17·8% [15·0–20·9%]) |
| Above sex-specific adult 99^th^ percentile | 51 (9·2% [7·0–12·0%]) | | 40 (14·2% [10·3–18·8%]) | | 116 (17·3% [14·5–20·4%]) |
| Above sex-specific pediatric 99^th^ percentile | 74 (13·4% [10·7–16·5%]) | | 41 (14·5% [10·6–19·2%]) | | 154 (23·0% [19·9–26·4%]) |
| Above sex-specific pediatric 97·5^th^ percentile | 87 (15·8% [12·8–19·1%]) | | 51 (18·1% [13·8–23·1%]) | | 184 (27·5% [24·2–31·1%]) |
| Median troponin concentration [IQR] | 1ng/L [1-4ng/L] | | 5ng/L [4-8ng/L] | | 2ng/L [2-13ng/L] |
| Age |  | |  | |  |
| Less than 1 year | 36 | | 33 | | 166 |
| Above local reference limit | 23 (63·9% [46·2–79·2%]) | | 24 (72·7% [54·5–86·7%]) | | 109 (65·7% [57·9–72·8%]) |
| Above sex-specific adult 99^th^ percentile | 19 (52·8% [35·5–69·6%]) | | 24 (72·7% [54·5–86·7%]) | | 107 (64·5% [56·7–71·7%]) |
| Above sex-specific pediatric 99^th^ percentile | 29 (80·6% [64·0–91·8%]) | | 24 (72·7% [54·5–86·7%]) | | 123 (74·1% [66·7–80·6%]) |
| Above sex-specific pediatric 97·5^th^ percentile | 31 (86·1% [70·5–95·3%]) | | 27 (81·8% [64·5–93·0%]) | | 142 (85·5% [79·3–90·5%]) |
| Median troponin concentration [IQR] | 32ng/L [20-82ng/L] | | 64ng/L [14-240ng/L] | | 46ng/L [15-128ng/L] |
| 1 to 12 years | 85 | | 119 | | 196 |
| Above local reference limit | 22 (25·9% [17·0–36·5%]) | | 22 (18·5% [12·0–26·6%]) | | 11 (5·6% [2·8–9·8%]) |
| Above sex-specific adult 99^th^ percentile | 25 (29·4% [20·0–40·3%]) | | 23 (19·3% [12·7–27·6%]) | | 11 (5·6% [2·8–9·8%]) |
| Above sex-specific pediatric 99^th^ percentile | 26 (30·6% [21·0–41·5%]) | | 21 (17·6% [11·3–25·7%]) | | 16 (8·2% [4·7–12·9%]) |
| Above sex-specific pediatric 97·5^th^ percentile | 30 (35·3% [25·2–46·4%]) | | 34 (28·6% [20·7–37·6%]) | | 23 (11·7% [7·6–17·1%]) |
| Median troponin concentration [IQR] | 2ng/L [1-35ng/L] | | 4ng/L [3-8ng/L] | | 2ng/L [1-2ng/L] |
| Over 12 years | 903 | 382 | | 878 | |
| Above local reference limit | 40 (4·4% [3·2–6·0%]) | 23 (6·0% [3·9–8·9%]) | | 80 (9·1% [7·3–11·2%]) | |
| Above sex-specific adult 99^th^ percentile | 40 (4·4% [3·2–6·0%]) | 25 (6·5% [4·3–9·5%]) | | 79 (9·0% [7·2–11·1%]) | |
| Above sex-specific pediatric 99^th^ percentile | 51 (5·6% [4·2–7·4%]) | 21 (5·5% [3·4–8·3%]) | | 94 (10·7% [8·7–12·9%]) | |
| Above sex-specific pediatric 97·5^th^ percentile | 73 (8·1% [6·4–10·1%]) | 38 (9·9% [7·1–13·4%]) | | 135 (15·4% [13·1–17·9%]) | |
| Median troponin concentration [IQR] | 1ng/L [1-2ng/L] | 4ng/L [2-6ng/L] | | 2ng/L [2-3ng/L] | |

**Supplementary table 4:** Lower reporting limits at each site during the study period. Where cardiac troponin concentrations were below this limit, they were assigned a value of half this limit.

| **Year** | **Site A** | **Site B** | **Site C** | **Site D** |
| --- | --- | --- | --- | --- |
| 2013 | 10 ng/L | 10 ng/L | - | Raw value |
| 2014 | 10 ng/L | 10 ng/L/4 ng/L | 1 ng/L | Raw value |
| 2015 | 10 ng/L/3 ng/L | 4 ng/L | 1 ng/L | Raw value |
| 2016 | 3 ng/L | 4 ng/L | 1 ng/L | Raw value |
| 2017 | 3 ng/L | 4 ng/L | 1 ng/L | Raw value |
| 2018 | 3 ng/L | 4 ng/L | 1 ng/L | Raw value |
| 2019 | 3 ng/L | 4 ng/L | 1 ng/L | Raw value |
| 2020 | 3 ng/L | 4 ng/L | 1 ng/L | Raw value |
| 2021 | 3 ng/L | 4 ng/L | 1 ng/L/3 ng/L | Raw value |
| 2022 | 3 ng/L | 4 ng/L/3 ng/L | 3 ng/L | 4 ng/L |
| 2023 | 3 ng/L | 3 ng/L | 3 ng/L | 4 ng/L |

**Supplementary Table 5:** Sensitivity analysis of the number (proportion [95% confidence intervals]) identified with myocardial injury in children and young adults between 1 and 18 years of age, by cardiac troponin assay (excluding infants <1 year of age).

|  | **All assays** | **hs-cTnI** | **hs-cTnT** |
| --- | --- | --- | --- |
| Participants from 1-18 years | 8729 | 2062 | 6667 |
| Above local reference limit | 753 (8·6% [8·0–9·2%]) | 153 (7·4% [6·3–8·6%]) | 600 (9·0% [8·3–9·7%]) |
| Above sex-specific adult 99^th^ percentile | 817 (9·4% [8·8–10·0%]) | 155 (7·5% [6·4–8·7%]) | 662 (9·9% [9·2–10·7%]) |
| Above sex-specific pediatric 99^th^ percentile | 792 (9·1% [8·5–9·7%]) | 187 (9·1% [7·9–10·4%]) | 605 (9·1% [8·4–9·8%]) |
| Above sex-specific pediatric 97·5^th^ percentile | 1226 (14·0% [13·3–14·8%]) | 261 (12·7% [11·3–14·2%]) | 965 (14·5% [13·6–15·3%]) |

**Supplementary Figure 1:** Cardiac troponin concentrations stratified by age & sex. Diamond represents median cardiac troponin concentration (ng/L). Error bars represent 25^th^ -75^th^ percentiles.

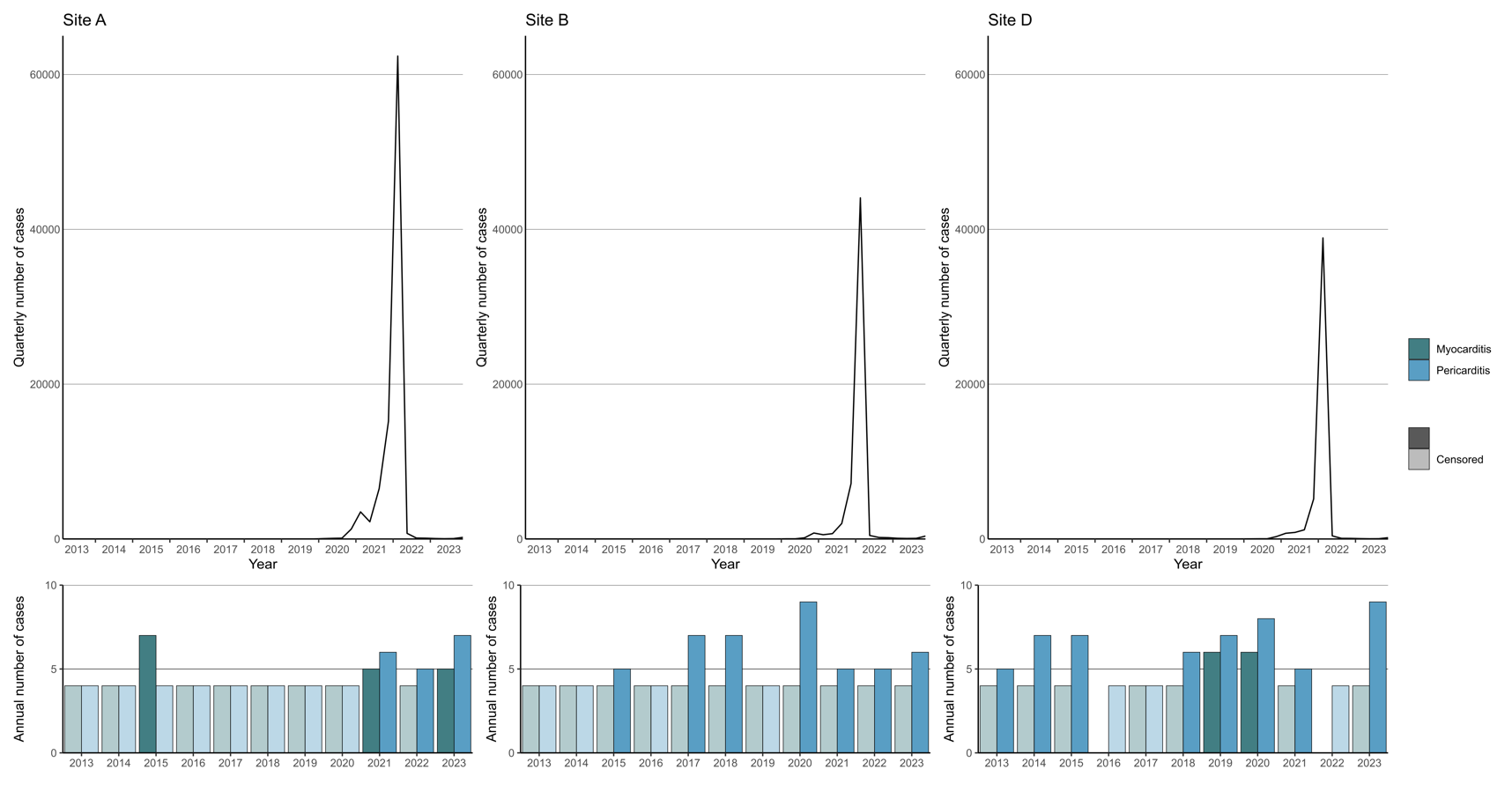
**Supplementary Figure 2:** Upper panel: Total regional COVID-19 cases in children (aged 0–19 years) at Norwegian sites, as recorded by the Notification System for Infectious Diseases from the Norwegian Institute of Public Health. Lower panel: Annual cases of myocarditis and pericarditis recorded at Norwegian sites in the Norwegian Patient Register. Where annual case numbers are between 1 and 4, the value is censored to protect individual patient identity.

**Supplementary Figure 3:** Upper panel: Quarterly hospital admissions with COVID-19 (IDC10 code U07·1) and vaccinations for COVID-19 in children (aged 0–18 years) at site C. Lower panel: Quarterly cases of pediatric multisystem inflammatory syndrome (ICD10 code U10·9), myocarditis and pericarditis recorded at site C in the Scottish Morbidity Record. As the case rate is below 5 per quarter, the data is presented as a density plot to protect individual patient identity.


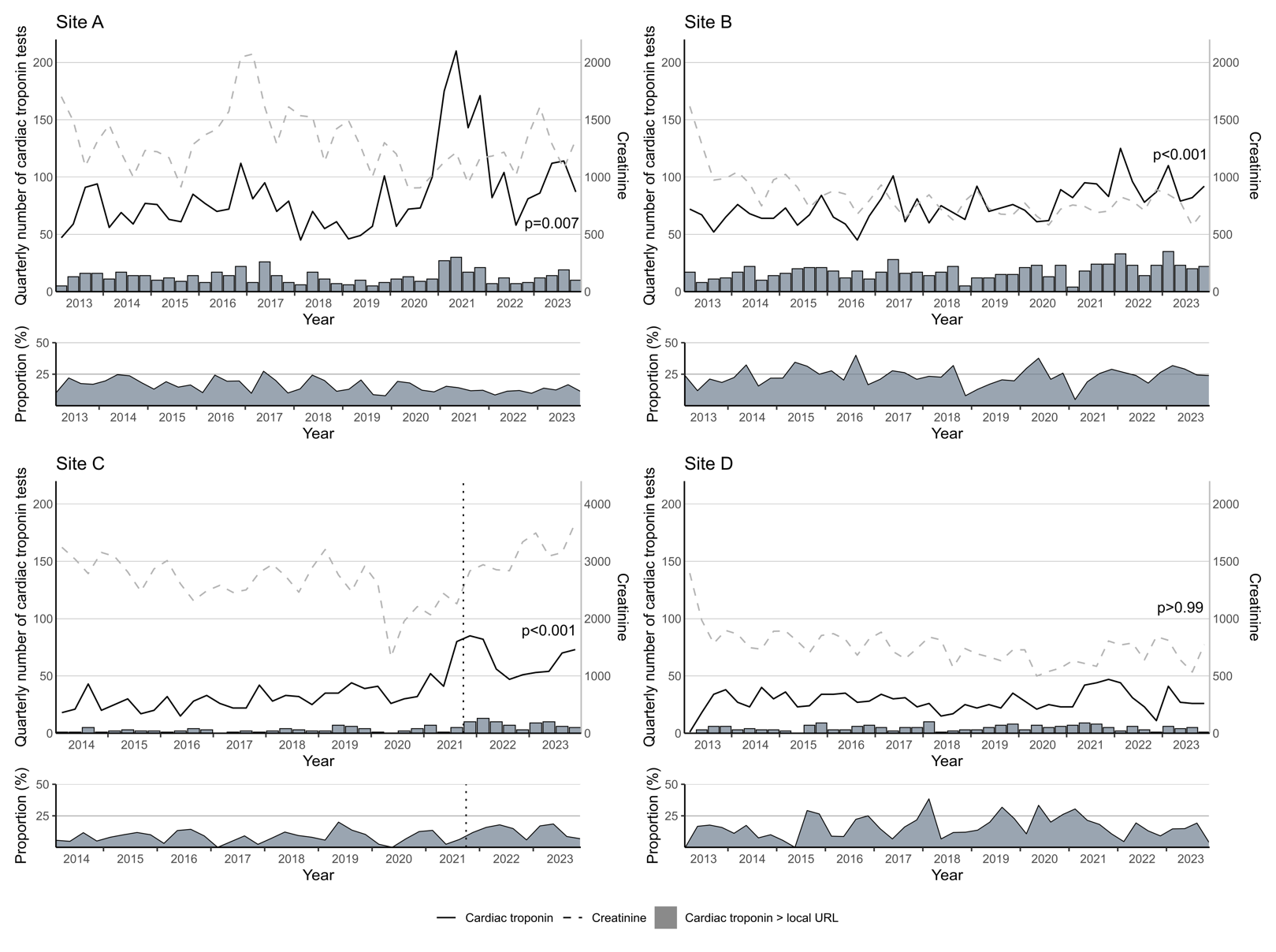
**Supplementary Figure 4:** Quarterly number of cardiac troponin tests, creatinine tests and proportion of tests above local URLs at each site. The lower panels For Site C, the vertical dashed line indicates the transition to high-sensitivity cardiac troponin T (hs-cTnT).

**Supplementary Figure 5:** Annual number of cardiac troponin tests and the number of tests exceeding the adult 99^th^ percentile, pediatric 99^th^ percentile and pediatric 97·5^th^ percentile at each site. The lower panels depict the proportion of tests above each threshold over time. For Site C, the vertical dashed line indicates the transition to high-sensitivity cardiac troponin T (hs-cTnT).


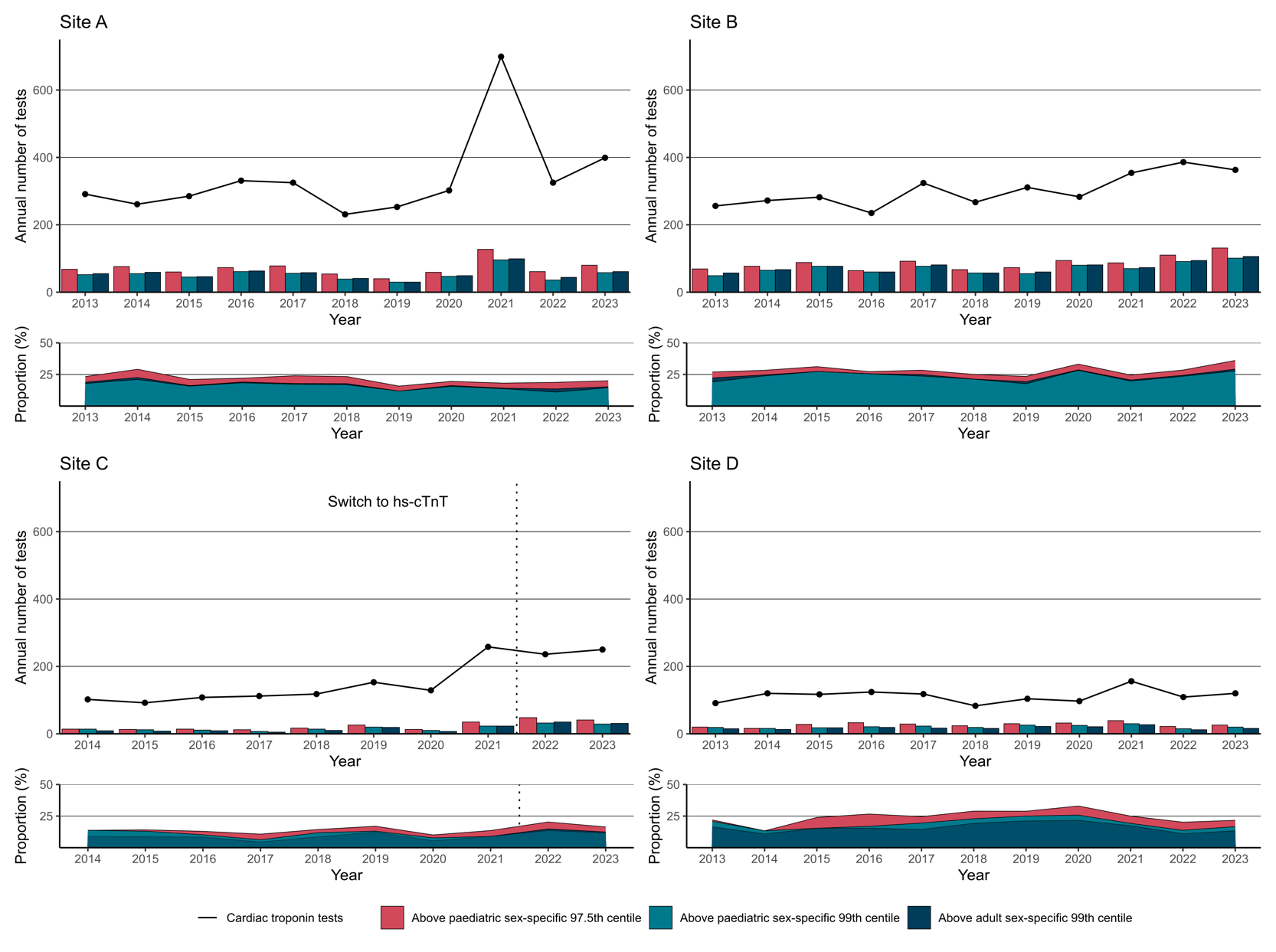

Supplement: jfaf205_Supplementary_Data [file jfaf205_supplementary_data.docx]
